# Scale up of fermentation of recombinant *Escherichia coli* for efficient production of spider drag silk protein MaSp1s and its dimers

**DOI:** 10.1186/s12934-025-02734-9

**Published:** 2025-05-14

**Authors:** Yufan Huang, Bixia Zhou, Ziyang Chen, Yongqin Su, Cheng Cheng, Bingfang He

**Affiliations:** 1 School of Pharmaceutical Sciences, Nanjing Tech University, No. 30 Puzhu South Road, Nanjing, 211816 China; 2 College of Biotechnology and Pharmaceutical Engineering, Nanjing Tech University, No. 30 Puzhu South Road, Nanjing, 211816 China; 3 2011 College, Nanjing Tech University, No. 30 Puzhu South Road, Nanjing, 211816 China

**Keywords:** MaSp1s, Fermentation optimization, *Escherichia coli*, Heterologous expression

## Abstract

**Background:**

Spider dragline silk exhibits ultrahigh tensile strength and excellent ductility, making it one of the best-performing natural biomaterials. The major ampullate spidroin (MaSp1) has promising applications in the biomedical, chemical, and military industries owing to its good biocompatibility, biodegradability, and low immunogenicity. The generation of recombinant spidroin can significantly facilitate its scaled production but has several challenges, including the high cost of the downstream spidroin solubilization process and the resulting toxicity due to the use of organic solvents. Unlike common MaSp, MaSp short (MaSp1s) from *Cyrtophoramoluccensis* is a low-molecular-weight spidroin, lacking the typical repetitive sequences and long poly(A) motif. These features enable the heterologous production of soluble spidroin.

**Results:**

In this study, rMaSp1 and its dimer rMaSp1s-2Core were expressed in soluble form by introducing the SUMO fusion tag and the self-shearing peptide intein. To improve the yield of recombinant spidroin using shake-flask fermentation, response surface analysis was used to optimize the conditions. The yields of rMaSp1 and rMaSp1s-2Core were 218.9 and 95.76 mg/L, respectively. Subsequently, fermentation was scaled up in a 5 L fermenter after adding metal ions and other growth factors to the medium. The optimal inoculation amount, induction temperature, loaded liquid, and feeding strategy were explored. Finally, the yields of rMaSp1 and rMaSp1s-2Core reached 1,112.2 and 297.8 mg/L, respectively. Furthermore, the dimerization of rMaSp1 monomers was achieved by introducing disulfide bonds via exogenous cysteine residues in the C-terminal domain. The secondary structure and self-assembly of rMaSp1 were also analyzed.

**Conclusion:**

This study successfully addressed key challenges in recombinant spidroin production by employing fusion tags (SUMO and self-shearing peptide intein) to enable the soluble expression of rMaSp1 and its dimer rMaSp1s-2Core. The secondary structure and self-assembly analyses further contributed to our understanding of recombinant spidroin. These findings enable the large-scale production of spidroin and its potential applications in the biomedical, chemical, and military industries, overcoming previous hurdles related to the solubility and toxicity associated with downstream processing.

**Supplementary Information:**

The online version contains supplementary material available at 10.1186/s12934-025-02734-9.

## Background

Spider silk is composed of various spidroins produced via different glands of spiders, and it has distinct functions in their growth and reproduction [1]. The major ampullate (MaSS) silk secreted from the major ampullate gland forms the framework of spider webs [2]. It is a highly efficient natural material owing to its excellent tensile strength and ductility, as well as its ease of isolation and accessibility [3]. However, because of their low silk production and cannibalistic tendencies [4], spiders cannot be bred for large-scale silk-fiber production. Therefore, recombinant biotechnology is increasingly used to produce spidroin for applications such as spinning artificial silks [5–7].

The MaSS is primarily composed of the major ampullate spidroin (MaSp). MaSp is rich in alanine and glycine, with the combined content of these amino acids reaching up to 50%. The core repeat region of MaSp contains two characteristic motifs: a crystalline region formed by the oligo-alanine (A)_n_ (*n* = 4–15) motif and a flexible region formed by the glycine-rich GGX/GPGXX/GPGPX (X = Y, L, or Q) motif. The oligo-alanine (A)_n_ motif mainly forms anti-parallel β-sheets in the silk, which collectively form the crystalline region of spider silk. This region primarily contributes to the high tensile strength of spider silk. The number of repeat units of MaSp, composed of crystalline and flexible regions, typically reaches hundreds. Therefore, the molecular weight of natural MaSp is generally between 200 and 350 kDa [8, 9] and typically consists of at least two MaSp proteins, MaSp1 and MaSp2 [10]. The high molecular weight (HMW) of natural spidroin and the high GC content of its genes introduce issues, such as gene recombination and premature translation termination [11], causing further difficulties in heterologous expression, such as low yield and inclusion body formation. Thus, the expression of full-length HMW spidroin is challenging in eukaryotes and prokaryotes, limiting the application of artificial spider silks in various fields. Furthermore, the high content of specific amino acids (Ala + Gly accounting for approximately 50%) and the presence of (A)_n_ motifs in natural spidroin result in high GC content (up to 70%) in the encoding genes. This, in turn, leads to the formation of complex and stable mRNA secondary structures. Additionally, high specific amino acids contents can cause a shortage of corresponding tRNAs, leading to premature translation termination. Despite these challenges, researchers have made significant progress by extensively modifying cell factory components and performing metabolic reconstruction [12–14]. Hauptmann et al. proposed a method for synthesizing HMW spidroin (250kD) in plants via intein-based multimerization [15]. Although a multimeric mixture of 2–10 structural domains was obtained using this method, large homogeneous proteins could not be obtained. Using amino acid mutagenesis, Lin et al. introduced a disulfide linkage between the two C-terminal structural domains in recombinant spider eggcase silk protein to produce a larger covalently bound silk protein (18 kD) [16]. Grip et al. introduced an AA-CC mutation in the poly-Ala chimera in the core region of the spidroin, forming a disulfide bond, enabling the formation of larger fibers (∼ 70 kDa) [14, 17]. The self-splicing action of inteins was utilized to increase the molecular weight of recombinant spidroin, enhancing the interactions between and within peptide chains. This facilitated the preparation of HMW artificial recombinant spider silk fibers. Because of intein splicing, the recombinant protein can undergo multiple rounds of aggregation reactions within cells, reaching an ultra-HMW of 2.4 MDa. Although this significantly enhanced the strength of the interchain hydrogen bonds in the protein assembly, the tensile strength was below 400 MPa [18].

For the large-scale production of artificial protein fibers, the Rising team developed low-molecular-weight (LMW) water-soluble artificial spider silk proteins. The protein yields in *Escherichia coli* were increased using batch fermentation and process optimization [19]. They also developed a biomimetic spinning device that can automate the preparation of protein fibers using only aqueous buffers. However, the performance of the artificial fiber was inferior to that of natural spider silk, and the batch stability was poor, making it unsuitable for industrial applications [20].

Han et al. discovered a new MaSp1 protein in *Cyrtophoramoluccensis*, which differs from previously identified MaSp. The full-length protein has a short core region comprising 439 amino acids. The newly identified LMW protein (40 kD) is called MaSp1s [21]. Unlike MaSp, its repeat (Rep) domain lacks typical poly(A) repeats [22]. Thamm et al. constructed a biomimetic spidroin based on the Rep domains of MaSp1s and MaSp2 [23]. The repeating units of the MaSp1s sequence contain a shorter poly(A) and a small amount of GGX, and the protein has a high polar amino acid content. Surprisingly, the MW and mechanical properties of MaSp1s bionic spider silk, which has “obvious defects” in its amino acid composition, are equivalent to those of MaSp2-type bionic spider silk.

Furthermore, the soluble production of recombinant spidroin is particularly interesting because it may help avoid the stylization of recombinant spidroin by an organic solvent, for example, hexafluoroisopropanol, facilitating downstream separation operations and spinning. Unlike other MaSp1 proteins, MaSp1s does not contain so many repetitive units (up to 100 times), and its sequence uniqueness makes it less difficult to express heterologously. However, the scale-up fermentation of MaSp1s and high molecular weight recombinant silk proteins remains underexplored. Future research endeavors should prioritize enhancing the number of spidroin repeats to improve the mechanical properties of the resulting silk materials.

In this study, we explored the scale-up fermentation of MaSp1s using a 5 L fermenter. Additionally, we successfully dimerized MaSp1s to form the rMaSp1s-2Core using the following two methods: (1) directly expressing the double repetitive sequence of the core region in vivo and (2) introducing disulfide bonds at the C-terminal to form dimers in vitro. The yields of the MaSp1s monomer and dimer were significantly increased using scale-up fermenters, reaching 1112.2 and 297.8 mg/L, respectively, which were 5.08 and 3.11 times higher, respectively, than those obtained using shake-flask fermentation. Moreover, to our knowledge, the MaSp1s yield is the highest soluble titer reported in *E. coli*. Additionally, the self-assembly performance and secondary structure of the MaSp1s monomer and dimer were characterized after expression and purification. The MaSp1s dimer exhibits better self-assembly ability and higher β-sheet content than the monomer. This work establishes a scale-up of the fermentation strategy for the efficient soluble production of recombinant spidroin and offers a preliminary investigation of its potential use in industry.

## Methods

### Construction of the Sumo-Intein-rMaSp1s and Sumo-Intein-rMaSp1s-2Core expression system

**Fig. 1:**
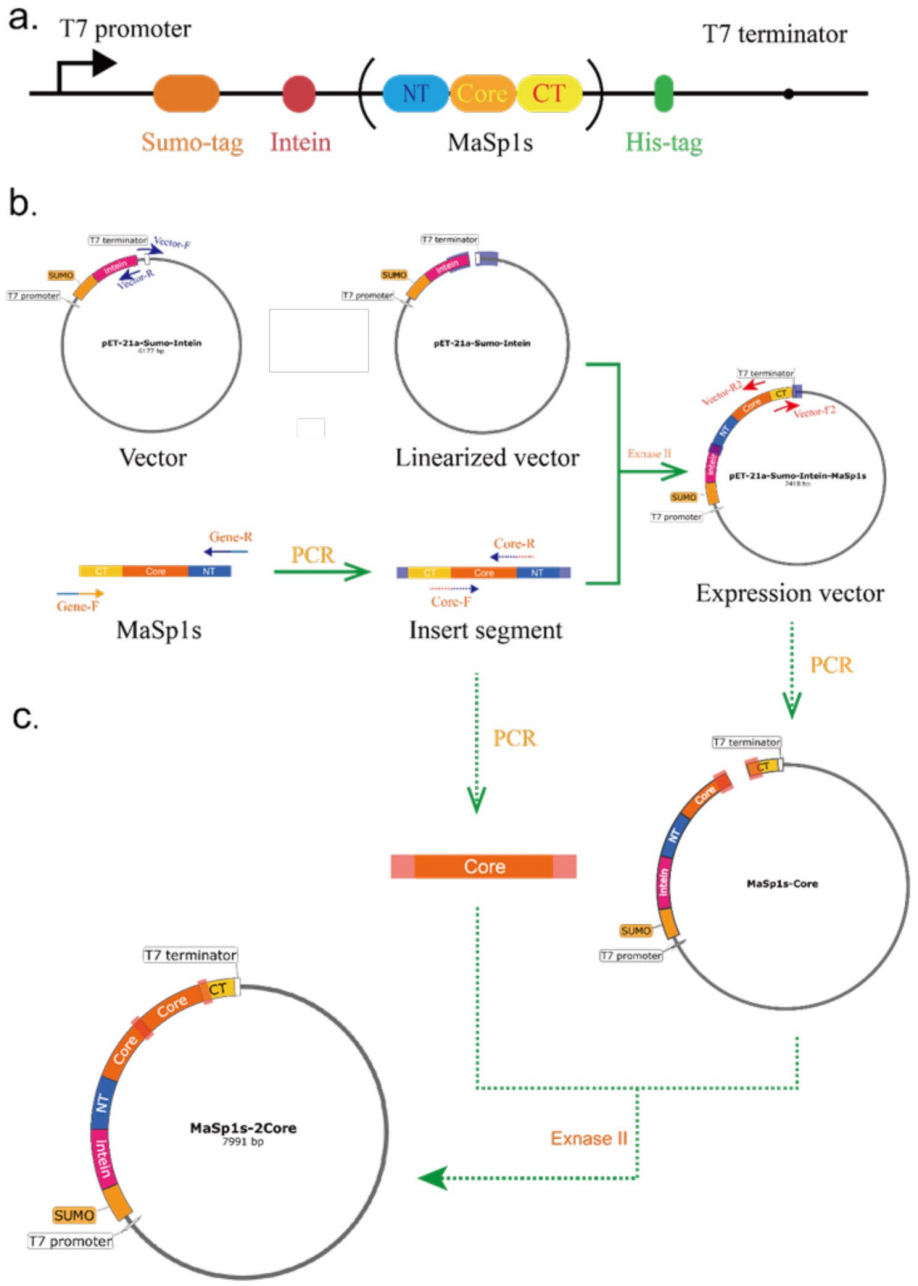
Schematic diagrams of recombinant expression system for rMaSp1s. (**a**) Sumo-Intein-rMaSp1s protein composition. Construction strategies for (**b**) Sumo-Intein-rMaSp1s and (**c**) Sumo-Intein-rMaSp1s-2Core

The bacterial strains, plasmids, primers, gene sequences, and respective references are listed in Table S1. The recombinant spidroin (rMaSp1s) was constructed based on MaSp1s from *Cyrtophoramoluccensis*, which contains only a short core region with typical conserved structural domains at both terminals. To achieve soluble expression of rMaSp1s, we constructed a recombinant expression system for fusion tag-intein-spidroin by introducing the Sumo fusion tag and an intrinsic intein peptide with self-cleavage function. The recombinant protein sequences also had a His-tag (green sequence) to enable further protein purification and confirmation (Fig. 1a). The pET21a (+)-Sumo-Intein-rMaSp1s expression vectors were constructed by combining the target gene fragments (rMaSp1s) with the pET21a (+)-Sumo-Intein vectors in a one-step cloning kit from Vazyme (Nanjing, China), as shown in Fig. 1b. Additionally, the subsequent rMaSp1s-2Core was constructed as described above (Fig. 1c). After the vector and inserted fragments were linearized, the recombination reaction was completed with Exnase II to achieve the in vitro cyclization of the linearized sequences. A single colony was selected for PCR identification and sequencing using the Genewiz sequencer (Suzhou, China) after the resulting recombinant plasmids were transformed into *E. coli* BL21(DE3) competent cells. Additionally, to obtain the MaSp1s dimer in vitro, we replaced the original terminal structural domains with those of *Latrodectus hesperus* MaSp1 (NRN1 and NRC1), which contains a cysteine residue at the 51st position at the C-terminus.

### Shake-flask fermentation optimization

The strain containing the recombinant expression plasmid was transferred to 30 mL of Luria–Bertani (LB) liquid medium containing 100 µg/mL ampicillin and incubated at 37 °C, 200 rpm until the OD_600_ was 0.6–0.8. Then, the incubator temperature was lowered to 25 °C, and 0.3 mM IPTG (working concentration) was added to the shaking flasks to induce protein expression for 18–20 h. Samples were obtained for shattering treatments and sodium dodecyl sulfate polyacrylamide gel electrophoresis (SDS-PAGE) verification. The optimal conditions for protein expression were explored by varying the inducer concentration (0.1, 0.2, 0.6, and 1 mM of IPTG), induction temperature (15 °C, 20 °C, 25 °C, 30 °C, and 35 °C), and induction duration (8, 12, 24, 36, and 48) for culturing *E. Coli* BL21 (DE3) cells containing the recombinant plasmid and an empty vector. Finally, the solubility ratio and yield by SDS-PAGE were used to identify ideal conditions for protein expression. The protein content in the total and soluble fractions was calculated using gray-scale scanning with Quantity One version 4.62 software (Bio-Rad, USA). The proteins were quantified using a BCA kit from Vazyme (Nanjing, China).

### Optimization of the culture conditions and medium composition

To identify the optimal conditions, the inducer concentration gradient was set at 0.2, 0.4, 0.6, and 0.8 mM, whereas the temperature gradient was 20 °C, 25 °C, 30 °C, and 37 °C. Then, a response surface analysis was performed to identify the optimal induction temperature, induction time, and the amount of inducer to be added using a design expert (Stat-Ease, Inc.) (Table 1), and all graphs were created using OriginPro (OriginLab, USA).

**Table 1:** Factors and levels of independent variables

Independent variables	Symbols	Range and levels		
		−1	0	+ 1
Induce temperature (°C)	A	20	25	30
Induce duration(h)	B	12	24	36
The concentration of IPTG (%)	C	0.2	0.4	0.6

Subsequently, the *E. coli* cells were cultured in 50 mL of fermentation medium (containing 10 g peptone, 20 g yeast extract, 10 g NaCl, 15.08 g NaH_2_PO_4_·2H_2_O, 6 g K_2_HPO_4_·3H_2_O, 43.75 mg MgSO_4_·7H_2_O, 0.64 mg FeSO_4_·7H_2_O, 1 mg CaCl_2_ per liter) in a 250 mL shake flask. The optical density at 600 nm (OD_600_) of the culture was measured using a UV spectrophotometer (Colibri, Inc.). The ratio of rMaSp1s to total protein was analyzed using the Gel-Pro Analyzer (Media Cybernetics, L.P.). Then, the concentrations of different carbon sources, including glucose, lactose, sucrose, maltose, and glycerin, were optimized to further enhance protein expression. Similarly, varying concentrations of nitrogen sources, including urea, Ala Gly, peptone, and yeast extract, were also explored.

### Scale-up fermentation of the Sumo-Intein-rMaSp1s and Sumo-Intein-rMaSp1s-dimer

Recombinant *E. coli* cells were cultured in a 5 L bioreactor (Winpact) containing 2 L fermentation medium. The fermentation medium contained 10 g peptone, 25 g yeast powder, 10 g NaCl, 8 g sucrose, 15.08 g NaH2PO4·2H2O, 6 g K2HPO4·3H2O, 43.75 mg MgSO4·7H2O, 0.64 mg FeSO4·7H2O, and 1 mg CaCl_2_ per liter. The seed culture was prepared by transferring the plate-detached strains into 100 mL of the fermentation medium and then into the 5-L bioreactor when the OD_600_ reached 5–6. The pH was maintained at 6.8 by adding 25% (v/v) ammonia during incubation. By adjusting the stirring speed, ventilation volume, and proportion of pure oxygen, the dissolved oxygen concentration was maintained at 25–40%. When the carbon source is depleted, the pH and dissolved oxygen concentration rise above the set value. The cells were fed at a certain rate with medium containing 400 g/L sucrose and 200 g/L peptone to maintain cell growth. Samples were collected and analyzed at regular intervals.

### Purification of the Sumo-Intein-rMaSp1s and Sumo-Intein-rMaSp1s-dimer

After washing the harvested cells 2–3 times with pure water, a portion of the washed cells was resuspended and placed on ice for sonication (power, 400 W; working time, 3 s; intermittent time, 5 s; total time, 10 min). The crushed solution was centrifuged at 4 °C, 13,523 × g for 3 min. The resulting samples were subjected to fixed-metal affinity chromatography purification using Ni^2+^-NTA agarose beads (QIAGEN) to obtain recombinant His-tagged Sumo-Intein-rMaSp1s [24]. The Ni-NTA column (3 mL) was pre-equilibrated with 10 column volumes of pure water and buffer A (containing 20 mM Tris, 140 mM NaCl) before adding the protein sample. After washing the impurities using 10 column volumes of buffer A, the column was rinsed with different gradients of buffer B (containing 100 mM imidazole, 20 mM Tris, and 300 mM NaCl) to obtain the eluate. Most of the rMasp1s was concentrated in the buffer B eluent at a concentration of 10% and then dialyzed using pure water. The dialysate was lyophilized and stored at − 80 °C.

The other portion of collected and washed cells was diluted to OD_600_ = 2–3. The cells were disrupted and centrifuged as described to separate the supernatant from the precipitate. Afterward, the precipitate was resuspended in pure water and mixed with 4 × protein loading buffer for SDS-PAGE analysis. The samples were boiled to denature the proteins and then electrophoresed on a 12.5% SDS-PAGE gel at 120 V. The gel was stained with Coomassie brilliant blue to visualize the bands. The amount of rMasp1s (% of total protein) was quantified using Gel-Pro Analyzer software (Media Cybernetics, 4.0.04). The total protein concentration was determined using the Bradford assay. The rMasp1s yield was calculated by dividing the mass of the purified lyophilized protein by the total protein mass.

### C-terminal dimerization of rMaSp1s and conditions optimization

The spidroin database was screened to identify the C-terminal residues of natural MaSp1s that can be replaced with cysteine to introduce disulfide bonds into the C-terminus and enable the formation of an HMW spidroin dimer. In the aqueous solution, only 15% of the proteins were dimerized spontaneously. To determine the ideal conditions for developing intermonomer disulfide bonds, we optimized the dimerization conditions considering the redox conditions and reaction duration. Purified MaSp1s protein was incubated at room temperature in conical tubes under varying ratios of GSSG and GSH. The mixtures were vortexed at 600 rpm for different durations to promote disulfide bond formation. After incubation, samples were collected and prepared using both reducing and non-reducing loading buffers for SDS-PAGE analysis. Different buffer conditions (Supplementary Table S2) were used to determine the optimal redox conditions.

### Secondary structure analysis

Circular Dichroism (CD): Recombinant spidroin conformations were determined using a JASCO J-1500 CD spectrometer in the far UV wavelength range of 185–260 nm. Then, a known concentration of the rMaSp1s monomer and dimer stock solution was diluted to 0.5 mg/L and subjected to Fourier transform infrared spectroscopy (FTIR) (Bruker OPTIK GmbH Tensor II, Germany) to analyze the secondary structure and the composition of the monomer and dimer. The rMaSp1s monomers and dimers were dried and subjected to spectroscopic determination using an attenuated total reflection detector with a scanning range of 4000–400 cm^− 1^, a resolution of 8 cm^− 1^, and 128 scans. The spectra were processed using Peakfit V 4.2 software. The Raman spectra in the amide I band (1700–1600 cm^− 1^) were fitted with second-order derivatives to obtain the content of each component by calculating the peak area.

### Self-assembly ability analysis

The morphologies of the two rMaSp1 nanofibers was observed using atomic force microscopy (AFM) (Bruker ICON). Before testing, the concentration of the chimeric spidroin stock solution was adjusted to 0.001% (w/v), and the diluted solution was subsequently dried dropwise on the surface of the mica flakes. Morphology was measured in scanning mode using a 2-nm radius probe with a mechanical modulus of 0.4 N/m. The final nanofiber morphology and mechanical data were analyzed using the in-built NanoScope Analysis 1.8 software.

## Results

### Soluble expression of Sumo-Intein-rMaSp1s and the Sumo-Intein-rMaSp1s-dimer

**Fig. 2:**
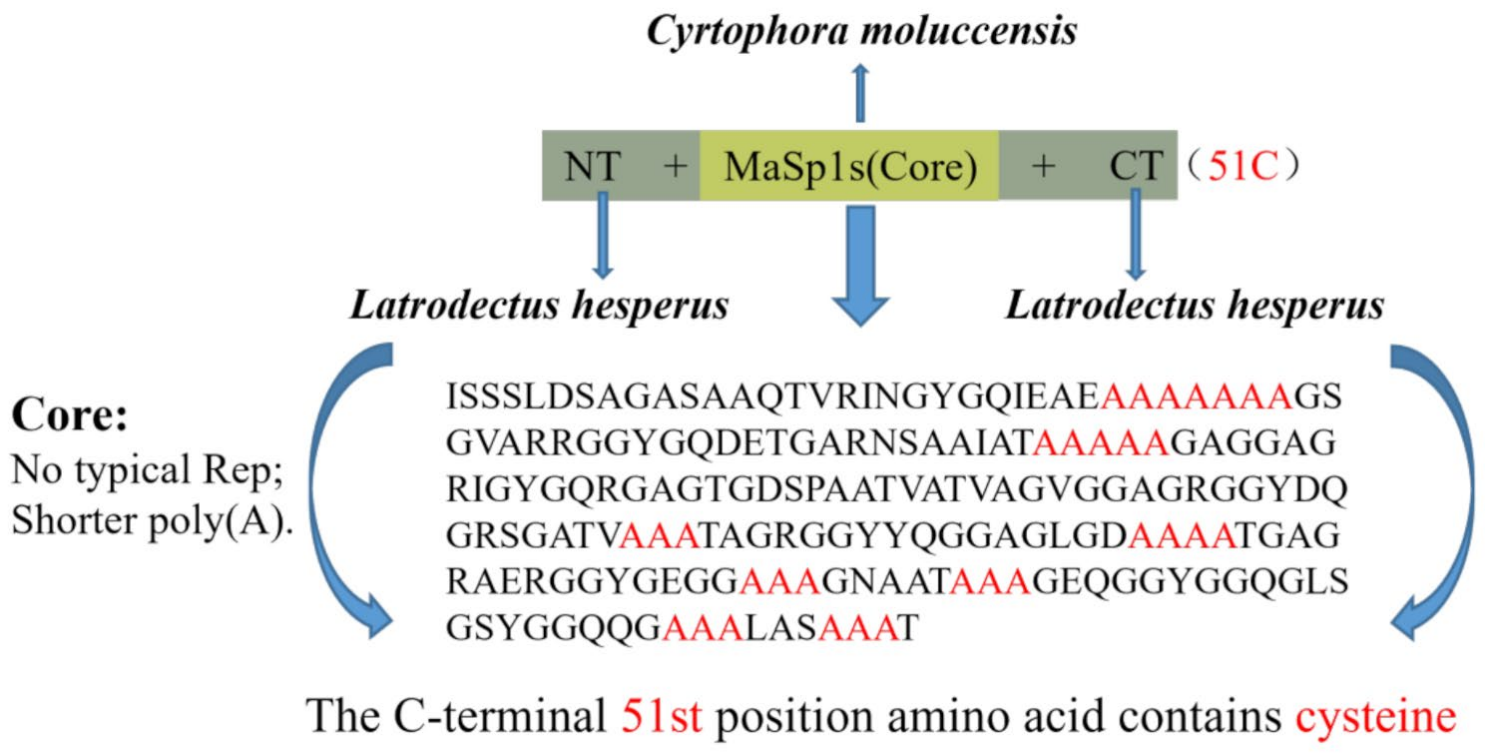
Structures of the gene and recombinant plasmid of rMaSp1s

MaSp1s from *Cyrtophoramoluccensis* have highly conserved terminal structural domains, similar to other MaSp1. The characteristic motifs in the core region of rMaSp1s are similar to those in MaSp1. However, it has a shorter ployA and lacks the typical repetitive region and proline residues (Fig. 2). To increase the molecular weight of the spidroin, disulfide bonds were strategically introduced into the Sumo-Intein-rMaSp1s monomer. Specifically, the natural C-terminal structural domains were replaced with the C-terminus of *L. hesperus* MaSp1s, which harbors a cysteine residue at the 51st position to enable disulfide-mediated rMaSp1s dimers (Fig. 2).

Furthermore, a recombinant expression system comprising fusion tag-intein-spidroin was constructed by introducing the Sumo fusion tag and short intein peptide with self-splicing ability. A histidine tag (6×His-tag) was added to the end of the gene for subsequent purification.

### Optimization of the expression conditions for Sumo-Intein-rMaSp1s

**Fig. 3:**
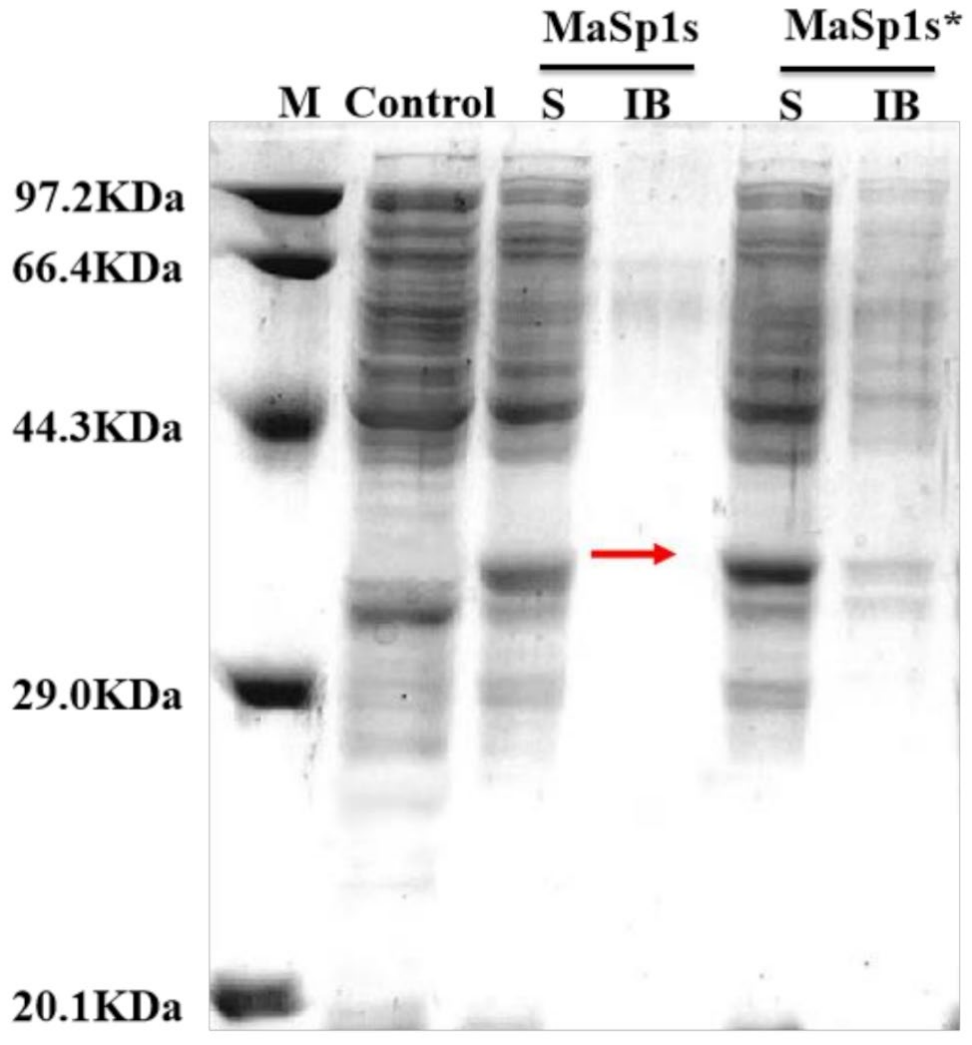
SDS-PAGE analysis of the cell culture samples before and after optimized induction conditions. MaSp1s and MaSp1s* indicate protein expression under conventional and optimized induction conditions, respectively. M: Protein marker; Control: Empty vector; S: Soluble fractions; IB: Inclusion bodies

rMaSp1s was successfully expressed in soluble *E. coli*. Although the theoretical MW of rMaSp1s is 42 kDa, the apparent MW observed on SDS-PAGE was 36 kDa (Fig. 3). This discrepancy is attributed to the unique structural properties of fibrous spidroin, which might alter its electrophoretic migration behavior.

The critical parameters, including the inducer concentration (IPTG), induction temperature, and duration, were systematically investigated to optimize the expression efficiency. Under optimized conditions, IPTG induction yielded predominantly soluble fusion proteins. The accumulation of by-products in *E. coli* gradually formed a micro-acidic environment, which facilitated the self-cleavage of the intein. As a result, the fusion proteins break down into Sumo-tagged intein and target protein (rMaSp1s). Optimization trials of the inducer concentration revealed that when the fusion protein was expressed using 0.3 mM IPTG, the spidroin was soluble and less likely to produce inclusion bodies (IBs). Consequently, 0.3 mM IPTG was deemed the optimal inducer concentration. As the induction time increases, IPTG induces cell rupture, enabling the release of the fusion protein into the culture medium. The number of IBs formed by the fusion protein increased at higher incubation temperatures. After optimizing the different conditions, the expression of soluble rMaSp1s was optimal at 35 °C, an IPTG concentration of 0.3 mM, and 24 h of induction. Under these conditions, recombinant protein expression constituted 15% of the soluble fraction. (Fig. 3)

**Fig. 4:**
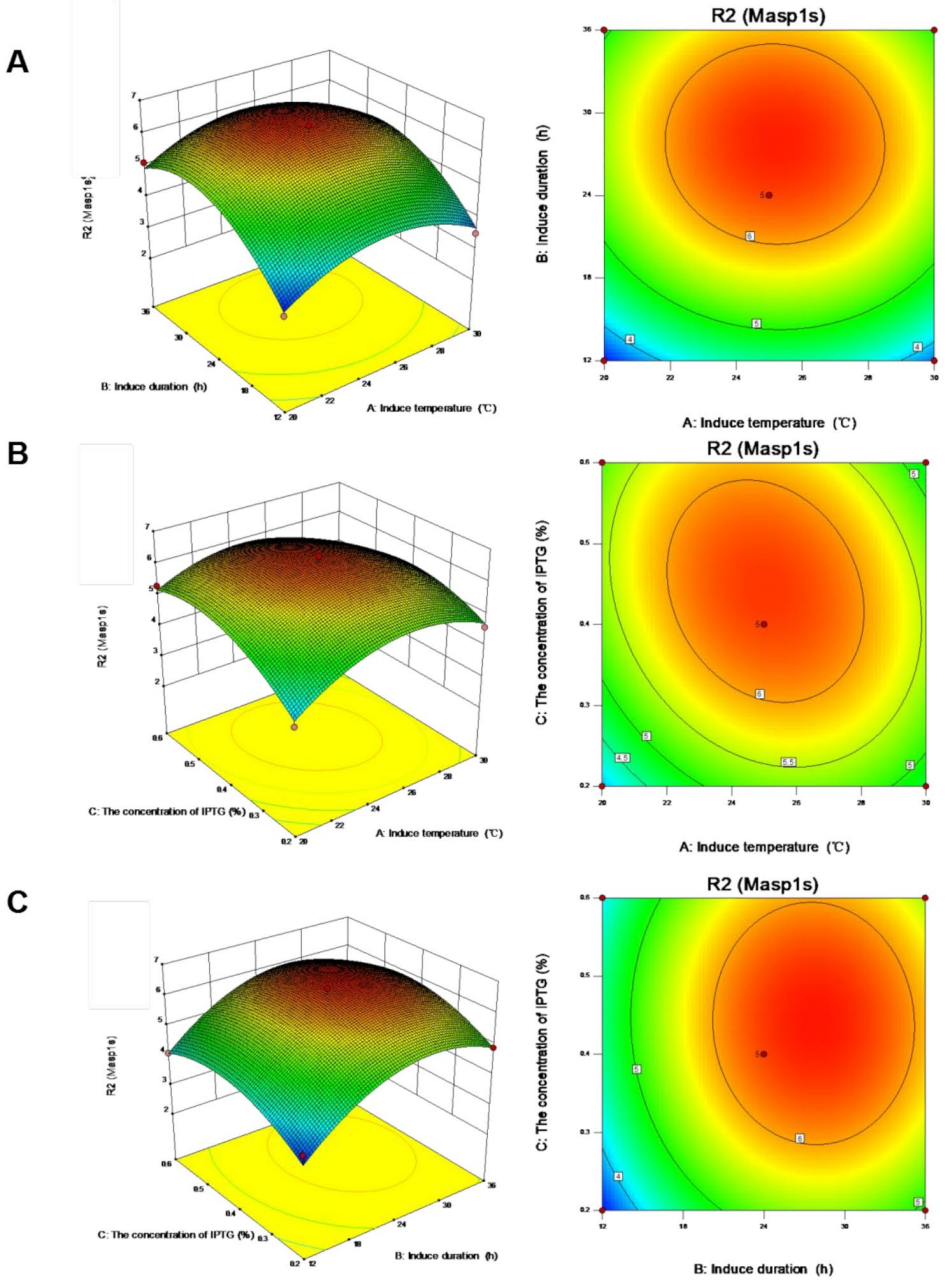
Effects of different growth conditions on rMaSp1s production. The conditions included (**a**) induction temperature, (**b**) induction time, (**c**) inducer concentration. Data are presented as mean ± standard deviation (*n* = 17)

Induction was performed in the early, middle, and late logarithmic growth phases. The SDS-PAGE results revealed that the total protein yield was higher in the early and middle stages. In the later stages of bacterial growth, metabolism is slowed, resulting in decreased protein expression. Therefore, cells were induced early during the logarithmic growth phase. To further optimize the growth conditions, we used response surface analysis to identify the induction temperature, induction time, and inducer amount required for rMasp1s production (Fig. 4). The results showed that when induction was performed at 20–30 °C for 24–36 h, the production of rMasp1s gradually increased, reached its highest value at 20–25 °C, and remained unchanged after 24 h. As shown in Fig. 4b, at an induction temperature of 25 °C and inducer concentration of 0.2–0.6%, the production of rMasp1s gradually increased, reached the highest value at 0.3–0.45%, and gradually decreased after 0.7%. Therefore, the inducer concentration and time were set at 0.4% and 12–36 h, respectively. rMasp1s production gradually increased and reached a maximum value at 18–25 h, then declined slightly (Fig. 4c). Finally, the predicted optimal induction conditions were a temperature of 25.29 °C, an IPTG concentration of 0.42%, and induction time of 26.25 h. The percentage of rMasp1s after verification was 9.63%.

**Fig. 5:**
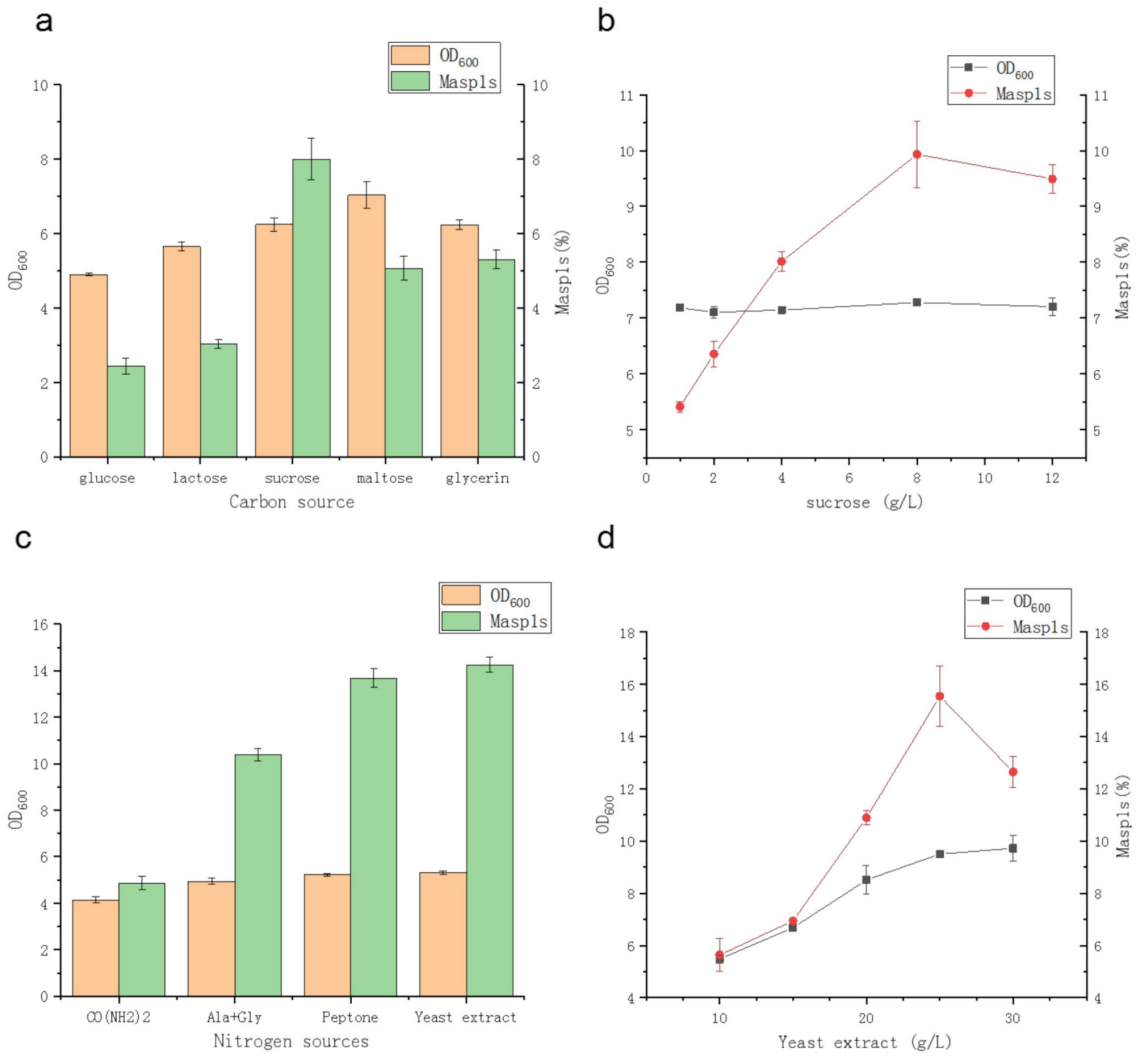
Effects of different carbon sources on MaSp1s expression. The graphs show the effects of (**a**) different carbon sources, (**b**) different sucrose concentrations, (**c**) different nitrogen sources, and (**d**) yeast extract on MaSp1 expression. All data was presented as mean ± SD (*n* = 3)

We also optimized the amounts of carbon and nitrogen sources added to the culture medium. rMasp1s production was highest in the culture medium when sucrose was used as the carbon source (adding the same mole number of carbon atoms), at 8.5% (Fig. 5a). When an additional 8 g/L of sucrose was added, the yield of rMasp1s reached 9.93% (Fig. 5b), which remained unchanged when the added sucrose exceeded 8 g/L. Compared with urea, the use of yeast powder and peptone as organic nitrogen sources significantly increased rMasp1s production (Fig. 5c). Among them, yeast powder had the best effect. With an additional 20 g of yeast powder, the yield of rMasp1s reached 14.25%. When the amount of yeast powder was increased from 10 to 25 g/L, rMasp1s production gradually increased but decreased when the yeast powder content exceeded 25 g/L. This decrease may be due to a high carbon-to-nitrogen ratio and enhanced metabolic acid production. Therefore, the amount of yeast powder was set at 25 g/L.

### Scale-up fermentation in bioreactors

Fermenters are widely used in microbial cultivation and protein production. The 5 L fermenter, an essential bioreactor, provides an ideal environment for scale-up fermentation. To further increase the rMaSp1s production, the culture conditions of the 5 L fermenter were adjusted based on the optimal conditions in a shake flask. During the logarithmic growth period, the bacterial density changes significantly, which in turn causes large pH fluctuations that may not be efficiently captured using a pH probe. Therefore, we added more NaH_2_PO_4_ and K_2_HPO_4_ to stabilize the pH. As shown in Fig. S1, the growth of recombinant *E. coli* entered the logarithmic phase after 6 h of cultivation, reaching the logarithmic metaphase after 12 h. The inducer was added simultaneously, and the cell density reached equilibrium after 24 h and then remained unchanged.

**Fig. 6:**
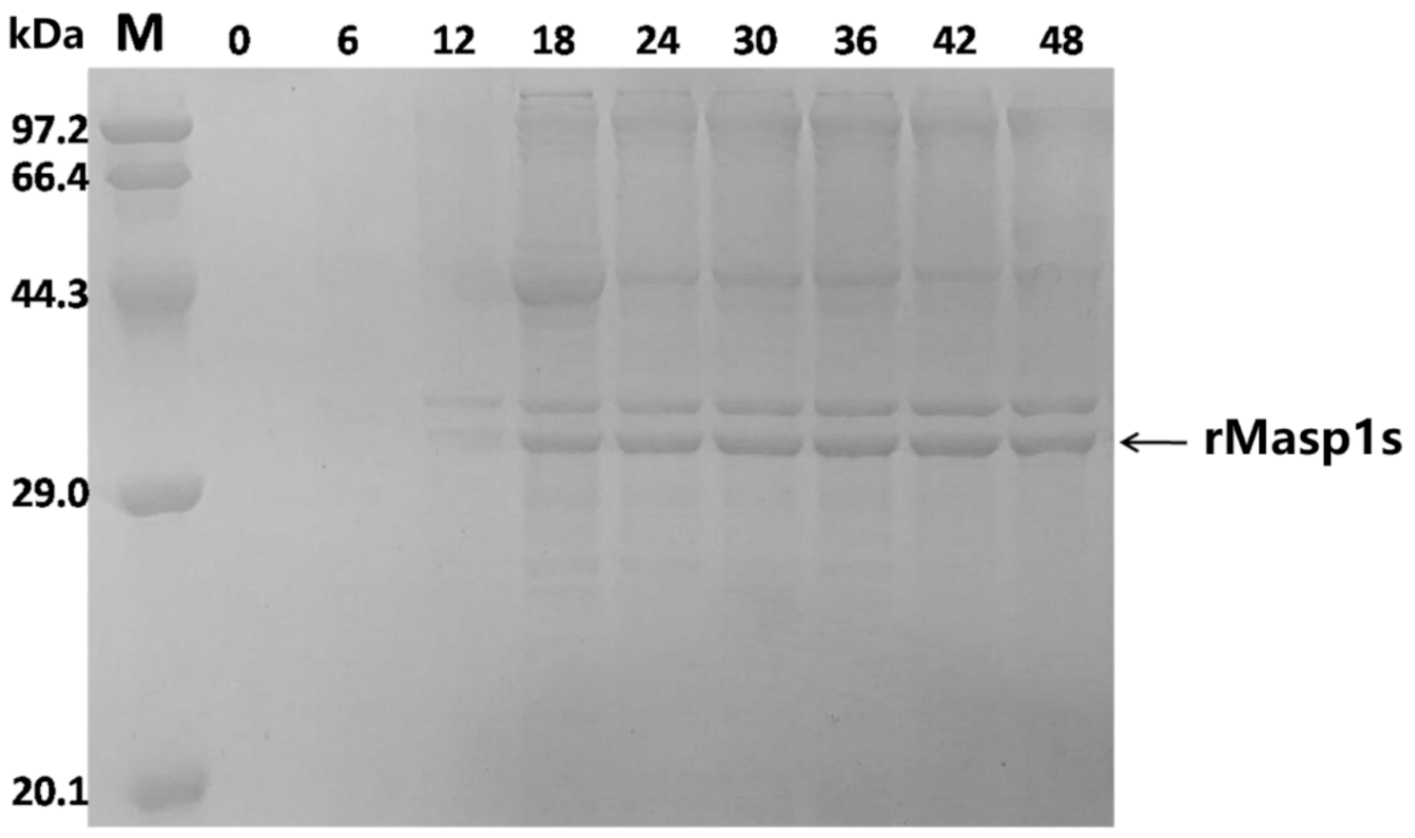
Effects of different induction periods on rMasp1s expression. The lanes show the protein marker (M), cell fraction before induction (0), and the fractions after induction for 6, 12, 18, 24, 30, 36, 42, and 48 h, respectively

**Fig. 7:**
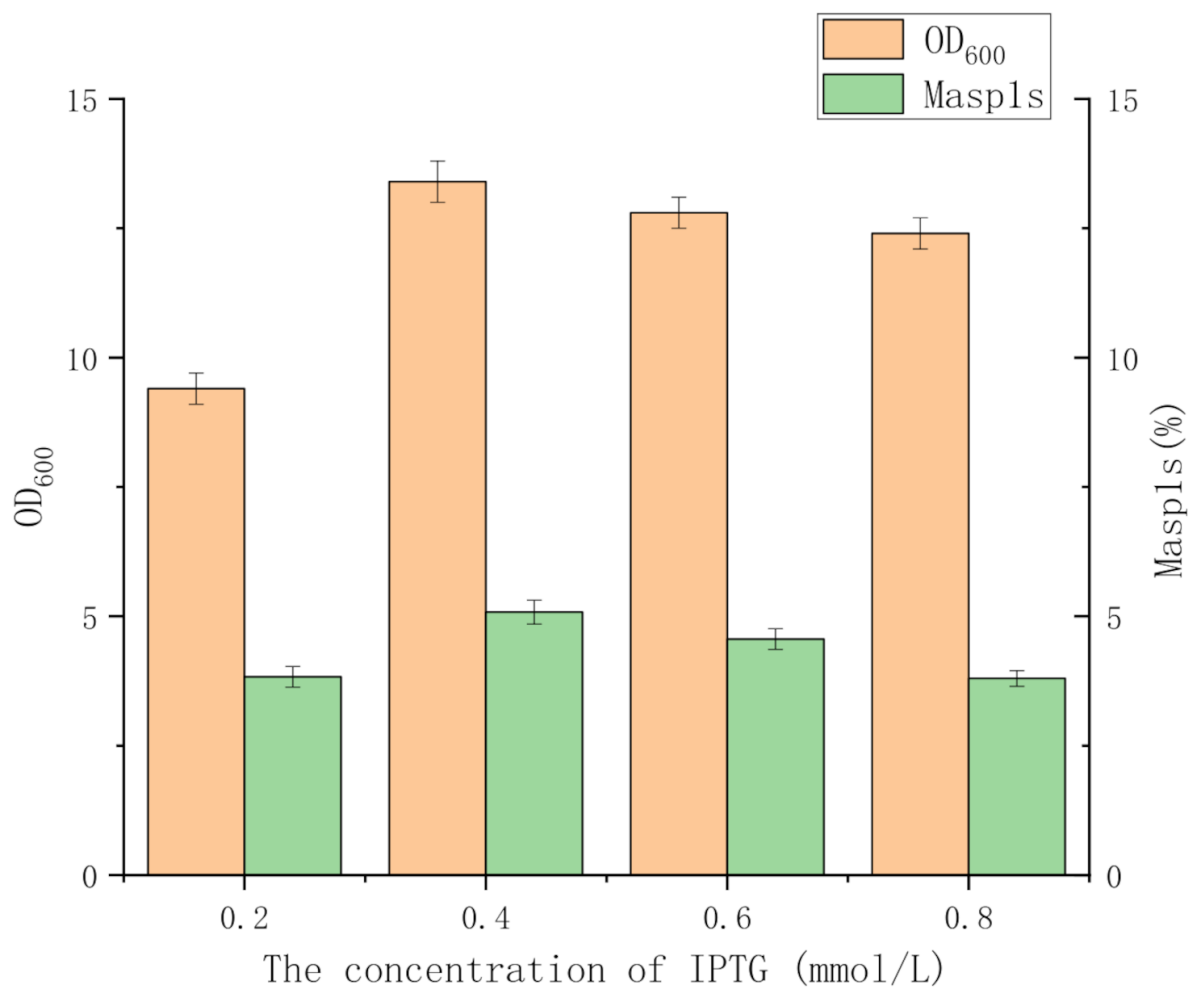
Effects of different IPTG concentrations on rMaSp1s expression.All data was presented as mean ± SD (*n* = 3)

When the fermentation time was extended, rMaSp1s production increased rapidly and reached a peak 36 h after the addition of the inducer, and then declined slightly at 48 h (Fig. 6). Additionally, optimization trials of the inducer concentration and induced temperature revealed that the rMaSp1s yield was highest at an inducer concentration of 0.4 mM (Fig. 7). High inducer concentrations inhibited *E. coli* metabolism, ultimately reducing the rMaSp1s yield. Although engineered *E. coli* grew rapidly at 37 °C, more IBs were formed. Most of the spidroin was expressed in a soluble form when the induced temperature was between 25 °C and 30 °C, which was ideal for the subsequent processes.

### Protein dimerization and optimization of the conditions for protein dimerization

**Fig. 8:**
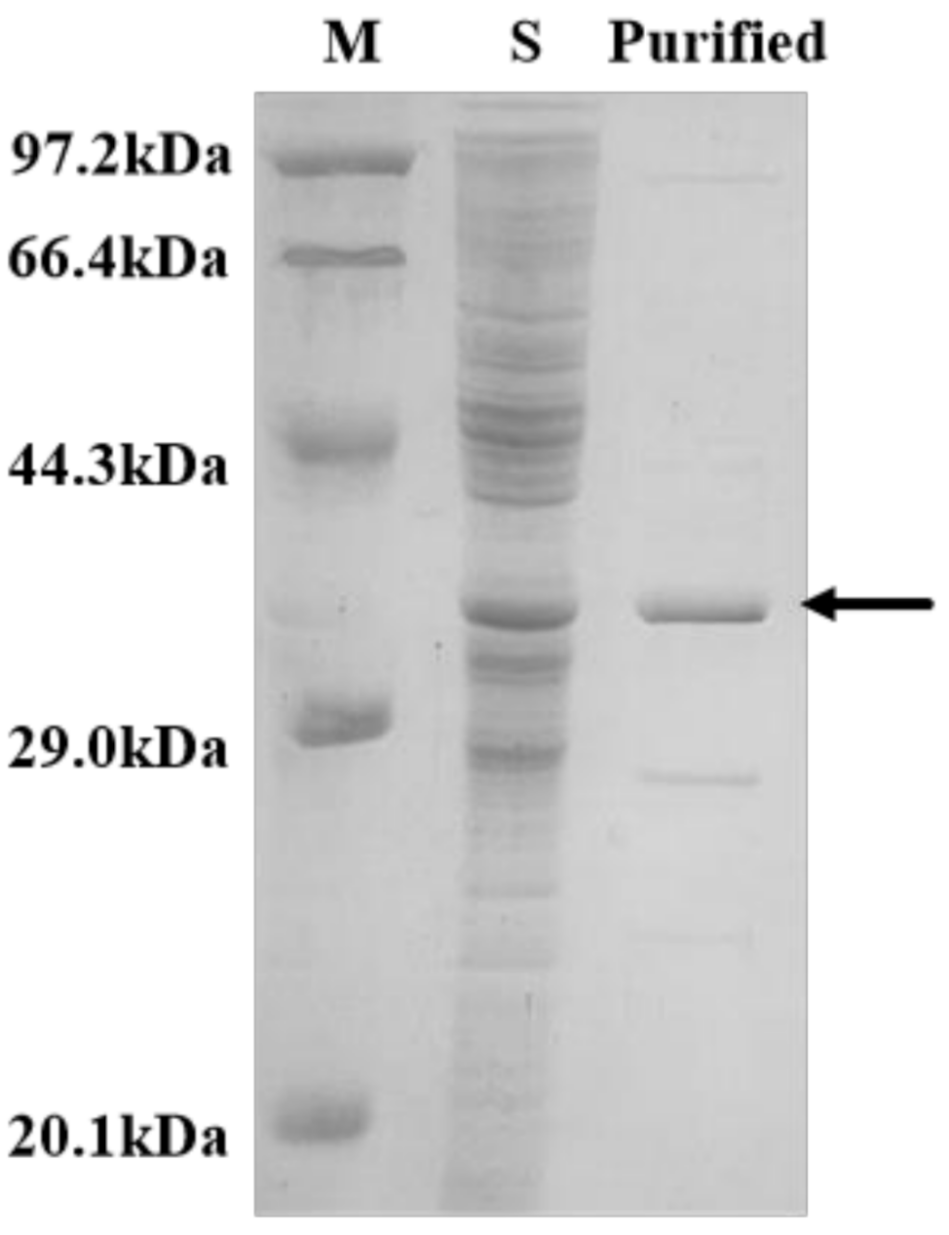
SDS-PAGE results for purified rMaSp1s. (M) protein marker; (S) fragmentation supernatant

**Fig. 9:**
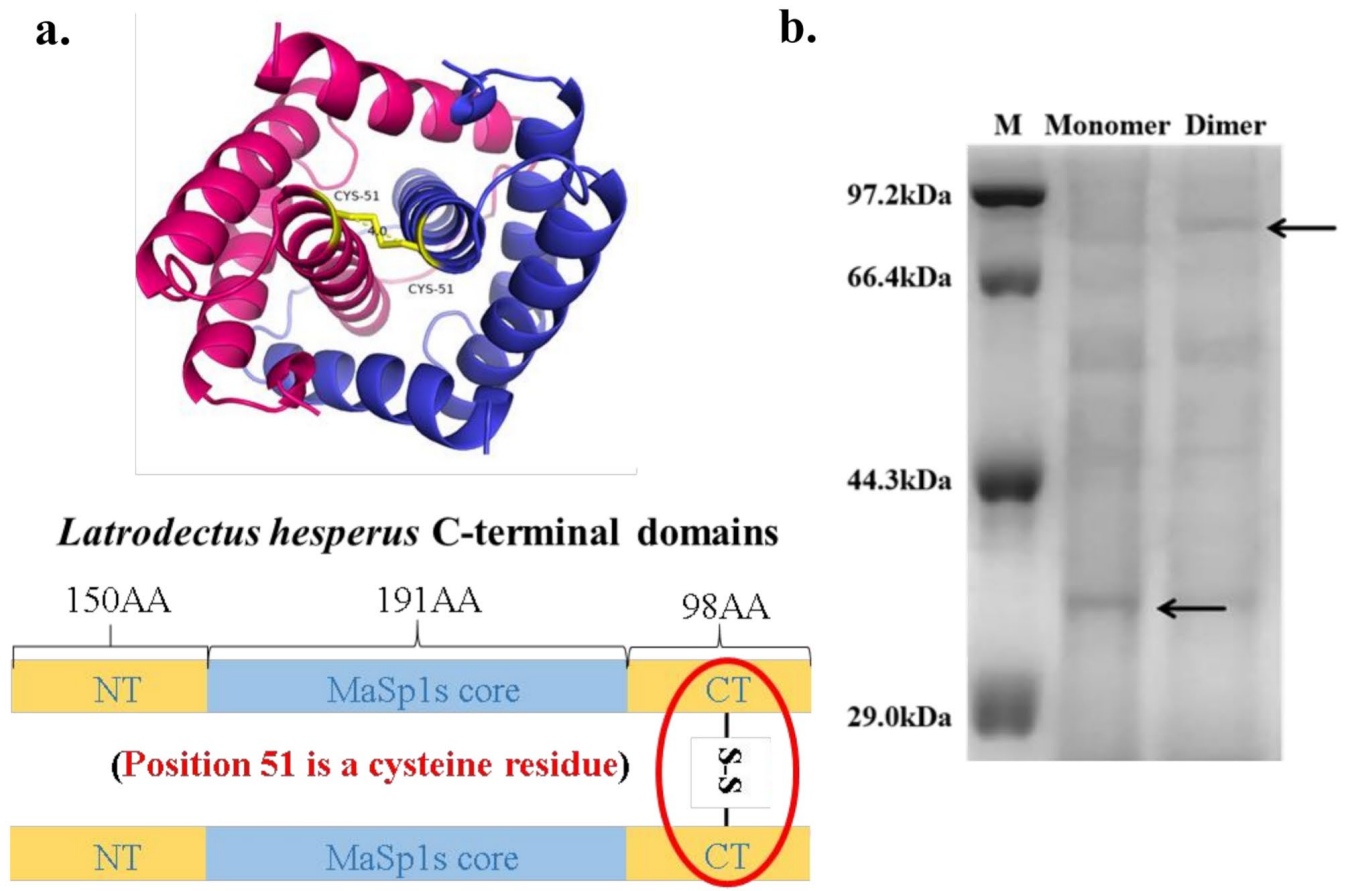
Schematic diagram of the dimerization of rMaSp1s. (**a**) The rMaSp1s C-terminal structural domain. (**b**) SDS-PAGE analysis of rMaSp1s before and after dimerization. Protein samples were prepared by mixing with sample buffer containing thiol-reducing agent, followed by heating at 100 °C for 5 minutes in a boiling water bath. The gel shows significant amounts of both dimer and monomer forms of rMaSp1s. The presence of reducing agents and heating may influence the observed band patterns

After successful expression and purification, rMaSp1s was obtained at a purity exceeding 95% using a 30 mM imidazole elution buffer (Fig. 8). The additional bands observed in the SDS-PAGE gel may arise from multiple factors. First, certain endogenous proteins in *E. coli* naturally contain histidine residues, which may co-purify with the target protein during Ni-NTA affinity chromatography [25]. Second, protein degradation during the purification process can generate smaller fragments that are also retained by the Ni-NTA resin, leading to the appearance of additional bands on the SDS-PAGE gel [26]. The yield (quantified using the BCA method) reached 190 mg/L. To facilitate subsequent functional studies, the purified protein was dialyzed overnight in water at 4 °C to exchange the buffer system. Further structural analysis revealed that *Latrodectus hesperus* MaSp1 contains cysteine residues at its C-terminus. Analysis of the PDB structure showed that the distance between the cysteines in the two C-terminal structural domains allows the formation of an interchain disulfide bond, enabling C-terminal dimerization (Fig. 9a). This dimerization increases the molecular weight of the spidroin, resulting in highly tensile artificial silk.

As described previously, different buffer conditions were used to determine the optimal redox conditions. The results showed that disulfide bond formation was more significant in the presence of 0 mM reduced glutathione (GSH) and 7 mM oxidized glutathione (GSSG). The results of reducing and nonreducing SDS-PAGE of rMaSp1s showed that while rMaSp1s migrated as monomers under reducing conditions, it mostly migrated as dimers under nonreducing conditions (Fig. 9b). This confirmed the successful dimerization of the spidroin via the formation of disulfide bonds in the C-terminal domains.

### Secondary structure and rMaSp1s self-assembly

**Fig. 10:**
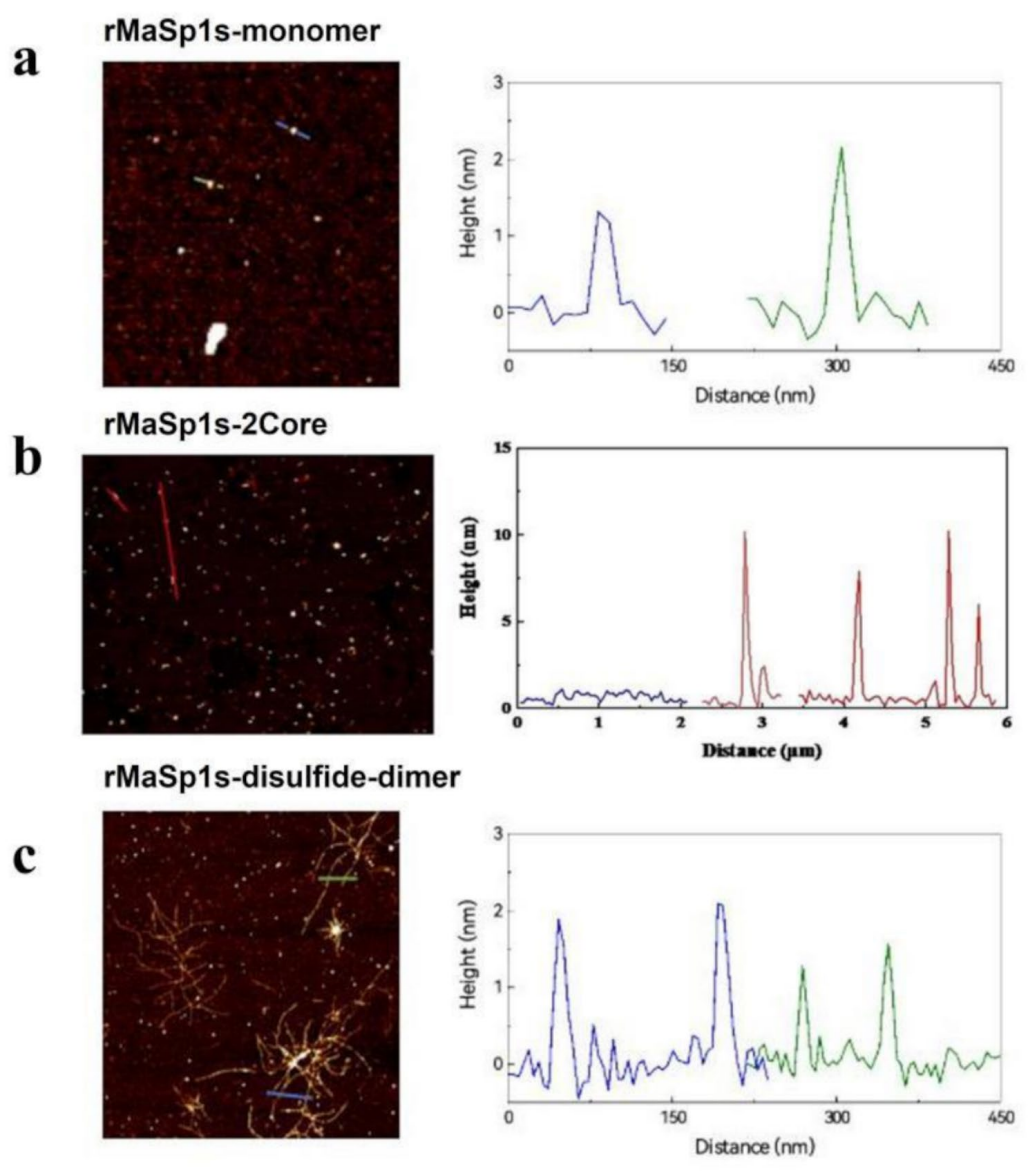
Atomic force microscopy results for the (**a**) rMaSp1s-monomer, (**b**) rMaSp1s-2Core, and (**c**) rMaSp1s-disulfde-dimer

The self-assembly of rMaSp1s-monomers, rMaSp1s-disulfide-dimer, and rMaSp1s-2Core was investigated using AFM. As shown in Fig. 10, the morphologies of the three forms of rMaSp1s differed significantly after 48 h of incubation at 60 °C. The rMaSp1s-monomers could barely form nanoprotofibril (Fig. 10a), possibly because of the LMW protein (only 191 amino acids in the core region). Under these conditions, the initial nanoparticles of the monomers were slightly aggregated into granules. rMaSp1s-2Core primarily assembles into irregular aggregates, initially forming molecular layers (Fig. S7) that further coalesce into particles with the height of 5–10 nm, while failing to form fibrous structures (Fig. 10b). Interestingly, the rMaSp1s-disulfide-dimer formed network-like nanofiber structures under the same incubation conditions (Fig. 10c). The diameter of these nanofibers is approximately 225 nm.

## Discussion and conclusion

MaSp1s is an LMW protein that lacks the repetitive region in the core structural domain and the typical long poly(A) [21]. Compared with common MaSp1, MaSp1s exhibits apparent deficiencies in amino acid composition and molecular weight. However, the mechanical properties of the bionic spider silks produced using MaSp1s were comparable to those made using MaSp2. Common MaSp forms IBs, which are difficult to dissolve. Furthermore, organic solvents, such as hexafluoroisopropanol, are required to dissolve heterologously expressed MaSp. This MaSp can be converted into spidroin solutions for use in spinning. The MaSp1s sequence reduces the difficulty of heterologous expression, which benefits soluble production. Therefore, further studies on MaSp1s were conducted.

As mentioned in a previous study [27], monitoring the intracellular environment and providing immediate feedback are the main challenges facing high-density spidroin fermentation. We are currently optimizing the detection and feedback methods of the reactor with the aim of providing a material basis for the study of spidroin. This work describes the design and study of the fermentation scale-up for the MaSp1s monomer and dimer using *E. coli*. Compared with the shake-flask fermentation, the yields of the rMaSp1s monomer and dimer after fermentation optimization were increased by 5.08-fold and 3.11-fold to 1112.2 and 297.8 mg/L, respectively. This represents a significant improvement in the rMaSp1s yield, which is 3–4 fold higher than that previously reported [23]. The MaSp1s monomer and dimer were purified from the cell supernatant to 95% purity. The purified spidroin solutions were ultrafiltered in this study. Thus, the high purity and concentration of the spidroin produced in this study make it suitable for characterization and spinning processes without requiring organic solvents for solubilization.

Moreover, this is the first study that investigated the MaSp1s dimer by two methods, which include (1) doubling the core region of MaSp1s to construct rMaSp1s-2Core for expression in vivo and (2) replacing the natural C-terminal structural domains with that from *Latrodectus hesperus* containing cysteine residues in suitable positions to form dimers in vitro.

The dimerization conditions of MaSp1s were also explored. The secondary structures and self-assembly abilities of the monomers and dimers were also characterized. We found that its dimers have significantly better self-assembly ability and can form distinct nanofibers than the rMaSp1s monomers. Using the abundant sequence and structural data available, more rigorous structure-property relationships of MaSp1s can be investigated to guide the development of high-performance synthetic fibers inspired by spidroin. The mechanical properties of the rMaSp1s monomer and dimer should also be explored. This work provides important evidence that can enhance the efficiency and scale-up of fermentation and the future applications of spider silk proteins.

## Electronic supplementary material

Below is the link to the electronic supplementary material.

Supplementary Material 1

## Data Availability

No datasets were generated or analysed during the current study.

## References

[1] Gatesy J et al. Extreme diversity, conservation, and convergence of spider silk fibroin sequences. 2001;291(5513):2603–5. 10.1126/science.1057561 11283372

[2] Marlene A et al. Morphology and Composition of the Spider Major Ampullate Gland and Dragline Silk. 2013. 10.1021/bm400898t 23837699

[3] Ko FK, Jovicic JJB. Model Mech Prop Struct Des Spider Web. 2004;5(3):780–5. 10.1021/bm0345099 15132661

[4] Wise DH. J.A.r.o.e., Cannibalism, food limitation, intraspecific competition, and the regulation of spider populations. 2006;51(1):441–65. 10.1146/annurev.ento.51.110104.150947 16332219

[5] Poddar H, Breitling R, Takano EJEB. Towards Eng Prod Artif Spider Silk Using Tools Synth Biology. 2020;4(1):1–6. 10.1049/enb.2019.0017 PMC9996717 36970229

[6] Rising A et al. Spider silk proteins: recent advances in recombinant production, structure–function relationships and biomedical applications. 2011;68:169–184. 10.1007/s00018-010-0462-z PMC11114806 20668909

[7] Bhattacharyya G et al. Large scale production of synthetic spider silk proteins in Escherichia coli. 2021;183:105839. 10.1016/j.pep.2021.105839 33746079

[8] Andersen S. O.J.C.b. And physiology. Amino Acid Composition Spider Silks. 1970;35(3):705–11.

[9] Sponner A et al. Differential polymerization of the two main protein components of dragline silk during fibre spinning. 2005;4(10):772–5. 10.1038/nmat1493 16184170

[10] Hinman MB, R.V.J.J.o.B C, Lewis. Isolation of a clone encoding a second dragline silk fibroin. Nephila clavipes dragline silk is a two-protein fiber. 1992;267(27):19320–4. 1527052

[11] Ayoub NA et al. Blueprint for a high-performance biomaterial: full-length spider dragline silk genes. 2007. 2(6): p. e514. 10.1371/journal.pone.0000514 PMC1885213 17565367

[12] An B et al. Inducing β-sheets formation in synthetic spider silk fibers by aqueous post-spin stretching. 2011;12(6):2375–81. 10.1021/bm200463e PMC3503542 21574576

[13] Zhang H et al. Microbial production of amino acid-modified spider dragline silk protein with intensively improved mechanical properties. 2016;46(6):552–8. 10.1080/10826068.2015.1084637 26460683

[14] Lewis RV et al. Expression and purification of a spider silk protein: a new strategy for producing repetitive proteins. 1996;7(4):400–6. 10.1006/prep.1996.0060 8776759

[15] Hauptmann V et al. Native-sized spider silk proteins synthesized in planta via intein-based multimerization. 2013;22:369–377. 10.1007/s11248-012-9655-6 23001519

[16] Lin Z et al. Engineered large spider eggcase silk protein for strong artificial fibers. 2013;25(8):1216–20. 10.1002/adma.201204357 23172740

[17] Grip S, Johansson J, Hedhammar MJPS. Eng Disulfides Improve Mech Prop Recombinant Spider Silk. 2009;18(5):1012–22. 10.1002/pro.111 PMC2771303 19388023

[18] Bowen CH et al. Recombinant spidroins fully replicate primary mechanical properties of natural spider silk. 2018;19(9):3853–60. 10.1021/acs.biomac.8b00980 30080972

[19] Schmuck B et al. High-yield production of a super-soluble miniature Spidroin for biomimetic high-performance materials. 2021;50:16–23.

[20] Andersson M et al. Biomimetic spinning of artificial spider silk from a chimeric Minispidroin. 2017;13(3):262–4. 10.1038/nchembio.2269 28068309

[21] Han L et al. Analysis of a new type of major ampullate spider silk gene, MaSp1s. 2013;56:156–161. 10.1016/j.ijbiomac.2013.01.034 23403024

[22] Spriestersbach A, et al. *Purification of his-tagged proteins*, in *Methods in enzymology*. Elsevier; 2015:1–15. 10.1016/bs.mie.2014.11.003 26096499

[23] Thamm C, Scheibel TJB. Recombinant production, characterization, and fiber spinning of an engineered short major ampullate spidroin (MaSp1s). 2017;18(4):1365–1372. 10.1021/acs.biomac.7b00090 28233980

[24] Zhang C et al. Engineered a novel pH-sensitive short major ampullate spidroin. 2020;154:698–705. 10.1016/j.ijbiomac.2020.03.153 32198037

[25] Gabe CM, Brookes SJ. and J.J.F.i.p. Kirkham, Preparative SDS PAGE as an alternative to his-tag purification of Recombinant amelogenin. 2017;8:424. 10.3389/fphys.2017.00424 PMC5472695 28670287

[26] Bornhorst JA, Falke JJ. *[16] purification of proteins using polyhistidine affinity tags*, in *Methods in enzymology*. Elsevier; 2000:245–54. 10.1016/s0076-6879(00)26058-8 PMC2909483 11036646

[27] Ruan C-R et al. Construction, fermentation and purification of high polymer spider dragline silk protein containing RGD peptide. 2007;23(5):858–61. 18051865

