# Supplementary material for: Scale up of fermentation of recombinant Escherichia coli for efficient production of spider drag silk protein MaSp1s and its dimers
Source: Microb Cell Fact. 2025 May 14;24:108. doi: 10.1186/s12934-025-02734-9 (PMC12080042; doi:10.1186/s12934-025-02734-9)
Supplement: Supplementary file 1 — Supplementary Material 1 [file 12934_2025_2734_MOESM1_ESM.docx]

**Supplementary material**

**Table S1**: Strains, plasmids, and primers used in this study.

| Strain/plasmid/Sequence/primer | Genotype or description/sequence | Definition |
| --- | --- | --- |
| Strains |  |  |
| *E. coli* DH5α | F-，φ80dlacZΔM15，Δ(lacZYA-argF) U169，deoR，recA1，endA1，hsdR17(rk-，mk+)，phoA，supE44，λ-，thi-1，gyrA96，relA1 | Host for cloning |
| *E. coli* BL21（DE3） | F-，ompT，hsdS（rBB-mB－），gal，dcm（DE3） | Host used for expression |
| Plasmids |  |  |
| pET21a | Novagen | Plasmid backbone |
| pET21a - SUMO - Intein | pET21a carrying the fusion tag SUMO and the Mini-Intein ∆I-CM gene | Synthesized by Genewiz (Suzhou, China). |
| pET21a-Intein-rMaSp1s | pET21a - SUMO - Intein carrying the rMaSp1s gene | This study |
| Sequences |  |  |
| NT | MTWSTRLALSFLLVLCTQSIYALAQANTPWSSKANADAFIN  SFISAASNTGSFSQDQMEDMSLIGNTLMAAMDNMGGRITPS  KLQALDMAFASSVAEIAASEGGDLGVTTNAIADALTSAFYQT  TGVVNSRFISEIRSLIGMFAQASAND | N-terminal domain from *Latrodectus hesperus* MaSp1 |
| CT | SALAAPATSARISSHASALLSNGPTNPASISNVISNAVSQISSSNP  GASACDVLVQALLELVTALLTIIGSSNIGSVNYDSSGQYAQVVT  QSVQNAFA | C-terminal domain from *Latrodectus hesperus* MaSp1 |
| Core | ISSSLDSAGASAAQTVRINGYGQIEAEAAAAAAAGSGVARRGG  YGQDETGARNSAAIATAAAAAGAGGAGRIGYGQRGAGTGDSP  AATVATVAGVGGAGRGGYDQGRSGATVAAATAGRGGYYQGG  AGLGDAAAATGAGRAERGGYGEGGAAAGNAATAAAGEQGGY  GGQGLSGSYGGQQGAAALASAAAT | Core region from *Cyrtophora moluccensis* MaSp1s |
| Intein | ALAEGTRIFDPVTGTTHRIEDVVGGRKPIHVVAAAKDGTLHA  RPVVSWFDQGTRDVIGLRIAGGAILWATPDHKVLTEYGWRAA  GELRKGDRVAQPRRFDGFGDSAPIPARVQALADALDDKFLHD  MLAEELRYSVIREVLPTRRARTFGLEVEELHTLVAEGVVVHNM | An intrinsic peptide, Mini-Intein ∆I-CM |
| Primers |  |  |
| Vector-F | CCCAAGCTTGCTCTGGCTGAAGGTACCCGTA | Upstream for amplification of pET21a - SUMO - Intein fragment |
| Vector-R | CCGCTCGAGGCCCACATACTGGCCAACGCTA | Downstream for amplification of NTRep2CT fragment |
| Gene-F | CTAGCTAGCGGTAATAGCGGCCGCGGTCA | Upstream for amplification of R2 fragment |
| Gene-R | CGGACTAGTACTATTACCACTCTGACCATAACCGC | Downstream for amplification of R2 fragment |

Note: Restriction sites are underlined in Table S1.

**Table S2**: Different buffer conditions for dimerisation reactions

| Spidroin type | Buffer conditions | Redox treatment | Reaction time |
| --- | --- | --- | --- |
| rMaSp1s | Reactions of spidroins in GSH/GSSG | No treatment | 0/12/24/36/48h |
|  |  | 0.3/3 mM |  |
|  |  | 3 mM/0.3 mM |  |
|  |  | 1.65 mM/1.65 mM |  |
|  |  | 0/5 mM |  |
|  |  | 0/7 mM |  |
|  |  | 0/9mM |  |


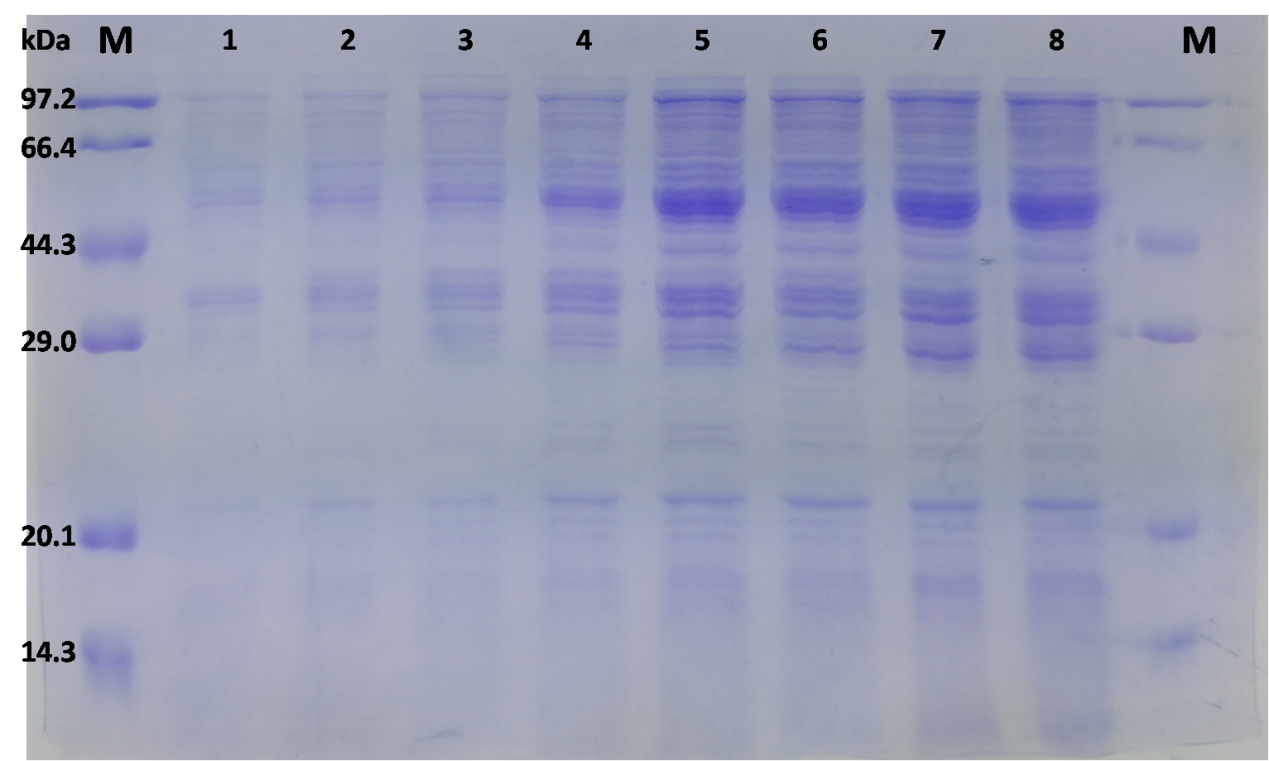


**Supplementary Fig. S1:** Effect of different induction lengths on Masp1s protein expression. M: protein marker; 1: without induction; induction for 3h(2),6h(3),12h(4),18h(5),24h(6),36h(7),48h (8)

**Supplementary Fig. S2:** Effect of the concentration of IPTG on Masp1s expression. All data was presented as mean ± SD (n=3).

**Supplementary Fig. S3:** Effects of different carbon sources on Masp1s expression. All data was presented as mean ± SD (n=3).

**Supplementary Fig. S4:** Effect of sucrose concentration on Masp1s expression. All data was presented as mean ± SD (n=3).

**Supplementary Fig. S5:** Effects of different nitrogen sources on Masp1s expression. All data was presented as mean ± SD (n=3).

**Supplementary Fig. S6:** Effect of the concentration of yeast extract on Masp1s expression. All data was presented as mean ± SD (n=3).


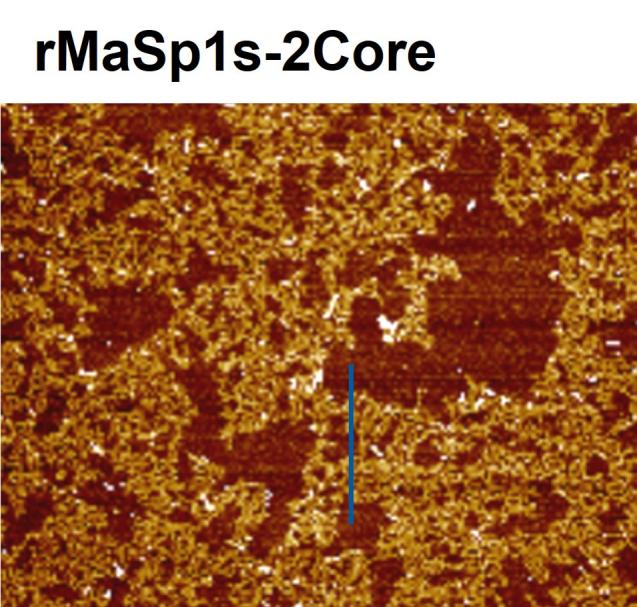


**Supplementary Fig. S7:** Atomic force microscopy analysis of fiber length in rMaSp1s-2Core.
